# Implications of the structure of human uridine phosphorylase 1 on the development of novel inhibitors for improving the therapeutic window of fluoropyrimidine chemotherapy

**DOI:** 10.1186/1472-6807-9-14

**Published:** 2009-03-16

**Authors:** Tarmo P Roosild, Samantha Castronovo, Michael Fabbiani, Giuseppe Pizzorno

**Affiliations:** 1 Department of Drug Development, Nevada Cancer Institute, Las Vegas, Nevada, USA

## Abstract

**Background:**

Uridine phosphorylase (UPP) is a key enzyme of pyrimidine salvage pathways, catalyzing the reversible phosphorolysis of ribosides of uracil to nucleobases and ribose 1-phosphate. It is also a critical enzyme in the activation of pyrimidine-based chemotherapeutic compounds such a 5-fluorouracil (5-FU) and its prodrug capecitabine. Additionally, an elevated level of this enzyme in certain tumours is believed to contribute to the selectivity of such drugs. However, the clinical effectiveness of these fluoropyrimidine antimetabolites is hampered by their toxicity to normal tissue. In response to this limitation, specific inhibitors of UPP, such as 5-benzylacyclouridine (BAU), have been developed and investigated for their ability to modulate the cytotoxic side effects of 5-FU and its derivatives, so as to increase the therapeutic index of these agents.

**Results:**

In this report we present the high resolution structures of human uridine phosphorylase 1 (hUPP1) in ligand-free and BAU-inhibited conformations. The structures confirm the unexpected solution observation that the human enzyme is dimeric in contrast to the hexameric assembly present in microbial UPPs. They also reveal in detail the mechanism by which BAU engages the active site of the protein and subsequently disables the enzyme by locking the protein in a closed conformation. The observed inter-domain motion of the dimeric human enzyme is much greater than that seen in previous UPP structures and may result from the simpler oligomeric organization.

**Conclusion:**

The structural details underlying hUPP1's active site and additional surfaces beyond these catalytic residues, which coordinate binding of BAU and other acyclouridine analogues, suggest avenues for future design of more potent inhibitors of this enzyme. Notably, the loop forming the back wall of the substrate binding pocket is conformationally different and substantially less flexible in hUPP1 than in previously studied microbial homologues. These distinctions can be utilized to discover novel inhibitory compounds specifically optimized for efficacy against the human enzyme as a step toward the development of more effective chemotherapeutic regimens that can selectively protect normal tissues with inherently lower UPP activity.

## Background

Uridine phosphorylase (UPP; EC 2.4.2.3) is a ubiquitous enzyme involved in pyrimidine salvage and maintenance of uridine homeostasis [1-3]. It catalyzes the reversible phosphorolysis of uracil ribosides and analogous compounds to their respective nucleobases and ribose-1-phosphate. The structural mechanisms underlying the catalytic activity of this enzyme have been extensively studied through analysis of *E. coli *UPP (EcUPP) [4-7] and more recently the *S. typhimurium *homologue [8]. These structures have shown UPP to belong to the nucleoside phosphorylase (NP) super-family of proteins in the NP-I subset of proteins possessing α/β folds and trimeric or hexameric (through trimerization of dimers) quaternary assemblies [9]. Additionally, based on conservation of sequence and ligand binding site architecture, it is probable that the general catalytic mechanism is retained between UPP and related purine nucleoside phosphorylases (PNPs).

Humans possess two isoforms of UPP (hUPP1 [10] & hUPP2 [11]) of which hUPP1 is more widely distributed, more abundantly expressed, and better characterized. hUPP1 has been a subject of interest to cancer researchers due to its role in the activation of pyrimidine nucleoside analogues used in chemotherapy, such as 5-fluorouracil (5-FU) [12] and its prodrug, capecitabine. Further, elevated levels of hUPP1 activity in certain tumours may contribute positively to the selectivity of these cancer-killing reagents [13]. Other studies have explored the potential of hUPP1 inhibitors as a means of raising endogenous uridine levels during the course of fluoropyrimidine nucleoside treatment, in order to protect normal tissues from the toxicity of these drugs [14,15]. These inhibitors have been developed from a family of acyclouridine analogues and include 5-benzylacyclouridine (BAU) [16], a compound that has been investigated in clinical trials for its ability to increase the therapeutic index of 5-FU through induction of such uridine-mediated rescue [17]. While structures of EcUPP with BAU and related molecular analogues have revealed the general mechanistic features of this competitive inhibitor which obstructs the enzyme's active site [7], the structure of hUPP1 and the details of its specific interactions with this potentially clinically-valuable drug have not been elucidated.

In the present study, we have determined the crystallographic structure of hUPP1 at high resolution, both in ligand-free and BAU-bound conformations (Table 1). The structures reveal significant global and local differences between the human enzyme and its microbial counterparts. This knowledge will be valuable in the future discovery and design of more potent and specific inhibitors of hUPP1 for development of improved chemotherapeutic regimens.

**Table 1:** Summary of crystallographic data and model refinement

**Diffraction Data**:				
Crystal Form	BAU		APO	
Source	SSRL 7-1		SSRL 9-1	
λ	1.00 Å		1.00 Å	
Space Group	P2_1_2_1_2_1_		F4_1_32	
Cell constants	a = 66.20 Å		a = 253.78 Å	
	b = 74.44 Å		b = 253.78 Å	
	c = 262.71 Å		c = 253.78 Å	
Mosaicity	0.40°		0.41°	
Resolution	50-1.90 Å	(1.97-1.90 Å)	50-2.3 Å	(2.38-2.30 Å)
Rmerge	11.9%	(44.1%)	7.2%	(38.4%)
I/σ	16.1	(2.0)	30.7	(5.7)
Completeness	99.8%	(100.0%)	99.9%	(99.7%)
				
**Model Refinement**:				
Number of reflections	97,957		30,108	
Number of monomers/A.U.	4		1	
Atoms/A.U.	9466		2396	
Protein	9030		2268	
Ligand	100		6	
Water	336		122	
Rcryst	20.5%		20.4%	
Rfree	25.1%		22.1%	
Rmsd bond lengths	0.018 Å		0.020 Å	
Rmsd bond angles	1.72°		1.68°	
Ramachandran statistics				
Most favored regions	91.5%		91.9%	
Additional allowed regions	8.5%		8.1%	

## Results

### hUPP1 is a dimeric enzyme

**Figure 1:**
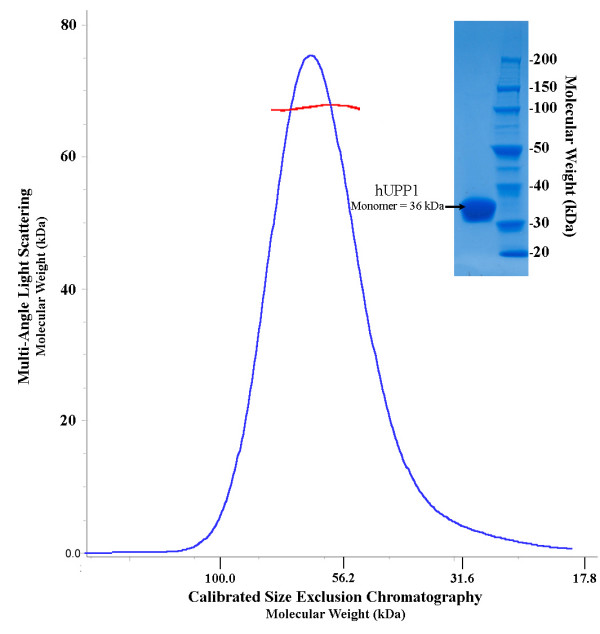
**hUPP1 is a dimeric enzyme**. In contrast to biophysically analyzed bacterial forms of uridine phosphorylase, human UPP1 is dimeric in solution as analyzed by both calibrated size-exclusion chromatography (blue; x-axis) and multi-angle light scattering analysis (red; y-axis).

Recombinantly expressed hUPP1 was noted to possess a smaller than expected quaternary assembly during purification by gel filtration. Analysis by both calibrated size-exclusion chromatography and multi-angle static light scattering (Figure 1) confirmed that the enzyme was dimeric in solution, in contrast to the hexameric assembly predicted from sequence analysis and protein family relationships [7]. Consistent with this observation, both solved crystal forms also yielded dimeric enzymes, verifying that the human form of this enzyme has lost the higher order secondary trimerization of dimers seen in microbial homologues.

### Overall structure of hUPP1

**Figure 2:**
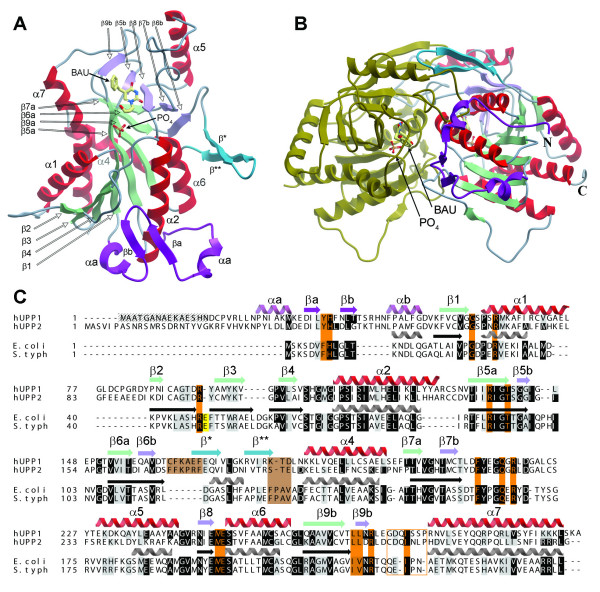
**Structure of hUPP1**. (A) The global fold of hUPP1 is conserved with its bacterial homologues with only slight modifications. The enzyme's N-terminus possesses additional architecture (purple) and the third helix in the *E. coli *protein (α3) is replaced by a sheet-turn-sheet structure (blue). All secondary structure is annotated so as to maintain consistency with prior microbial structures. (B) Shown is the dimeric biological unit for hUPP1. It is noteworthy that both of the aforementioned structural modifications (purple & blue) result in substantial increases in the buried surface area within the dimer interface of the enzyme. (C) Structure-based sequence alignment between the two human enzyme isoforms and structurally analyzed bacterial homologues reveals strict conservation of all of the residues lining the enzyme active site (orange) with the sole exception of a phenylalanine to tyrosine variation at the N-terminus of a residue that contacts primarily inhibitory molecules, such as BAU. There are also substantial differences in the loop lining the back of the ligand-binding pocket that similarly interacts only with inhibitor chemical groups and not natural substrates (boxed). Modifications underlying loss of dimer trimerization in hUPP1 are highlighted in brown. Interestingly, the glutamate implicated in bacterial enzymes in K^+ ^coordination at the dimer interface (yellow) is absent from both human proteins. Residues of hUPP1 that are disordered in both structures are hatched.

The overall global fold of hUPP1 is highly similar to its microbial UPP homologues and other members of the NP-I family of proteins, with two notable exceptions. First, there is substantial additional architecture at the N-terminus of the protein forming a strand-turn-strand structure bracketed by two short helices (Figure 2A). Second, the third α-helix of the bacterial enzymes (α3) has been replaced by an additional strand-turn-strand motif. While not directly impacting the structure of the active site of the enzyme, both of these changes increase the buried surface area of the dimer interface (Figure 2B). Quantitatively, these modifications together increase the interface area between subunits approximately 18%, from 2791 sq. Å in the *E. coli *enzyme to 3292 sq. Å in hUPP1.

The alterations occurring around the region of microbial helix α3 also provide a molecular explanation for the loss of trimerization of dimers in the human enzyme. The long loop preceding β* in hUPP1 sterically interferes with the formation of the protein-protein interface for dimer oligomerization seen in previous structures. Additionally, the hydrophobic residues ('FPAV') that form the core of the trimer assembly surface in EcUPP have been mutated to more polar, solvent-compatible residues in hUPP1 (Figure 2C).

**Figure 3:**
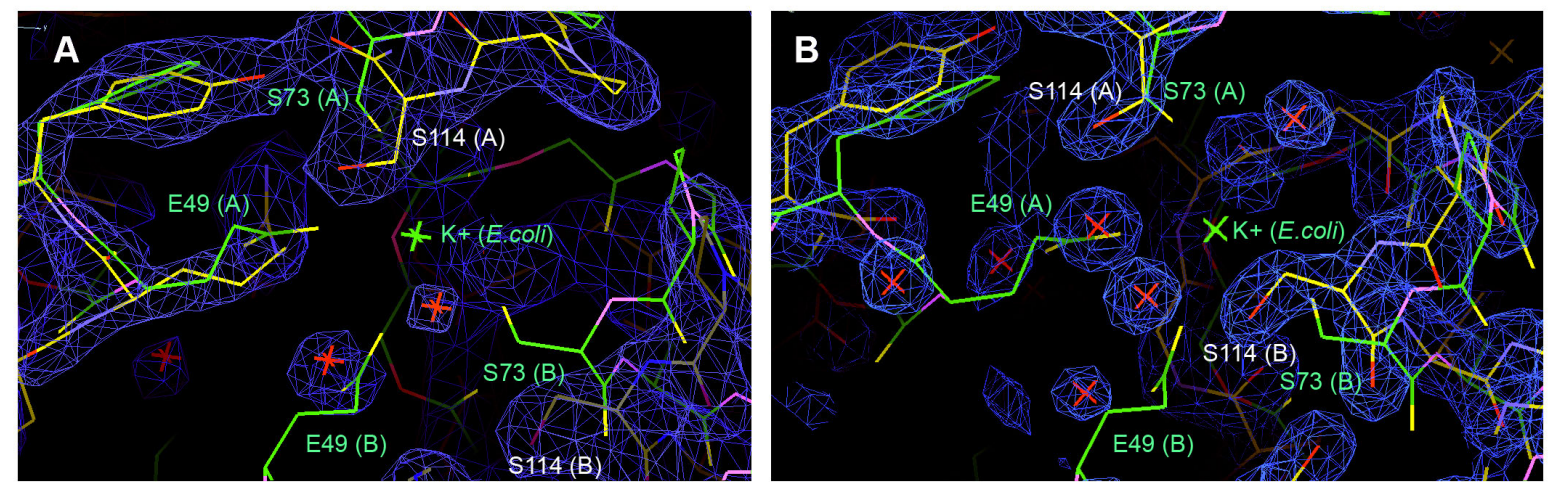
**Structure of the *E. coli *K^+ ^binding site in hUPP1**. (A) Structural alignment of the K^+ ^binding site of EcUPP (green) with the ligand-free structure of hUPP1 (yellow) reveals both a lack of density for K^+ ^and loss of the coordinating glutamate residues (E49). Comparison of the equivalent serine residues (S114 in hUPP1; S73 in EcUPP) shows that rather than binding K^+^, the human residues are turned away from the region and spread further apart by the global inter-domain motion observed for the ligand-free enzyme. The electron density for hUPP1 is shown (blue; 2f_*o*_-f_*c *_map contoured at 2σ) with crystallographic waters marked as red crosses. (B) Structural alignment of the K^+ ^binding site of EcUPP (green) with the BAU-bound structure of hUPP1 (yellow), shown from the same perspective as in (A), confirms that, despite its inclusion in all crystallization solutions, K^+ ^is not present at the dimer interface as previously observed in EcUPP.

Another difference comparing the hUPP1 structure to previously studied microbial counterparts is that the stabilizing K^+ ^binding site observed at the dimer interface of those proteins [6] is not occupied in either of the determined human structures (Figure 3). From structure-based sequence alignment it becomes clear that the primary coordinating residue (Glu49 in EcUPP) is deleted from the human proteins with surprisingly little distortion in the surrounding architecture (Figure 2C). That K^+ ^no longer plays a role in enzyme stabilization was unexpected, as high levels of salt needed to be maintained in all protein purification and crystallization buffers to prevent hUPP1 aggregation and precipitation.

### Binding of the inhibitor BAU to the hUPP1 active site

**Figure 4:**
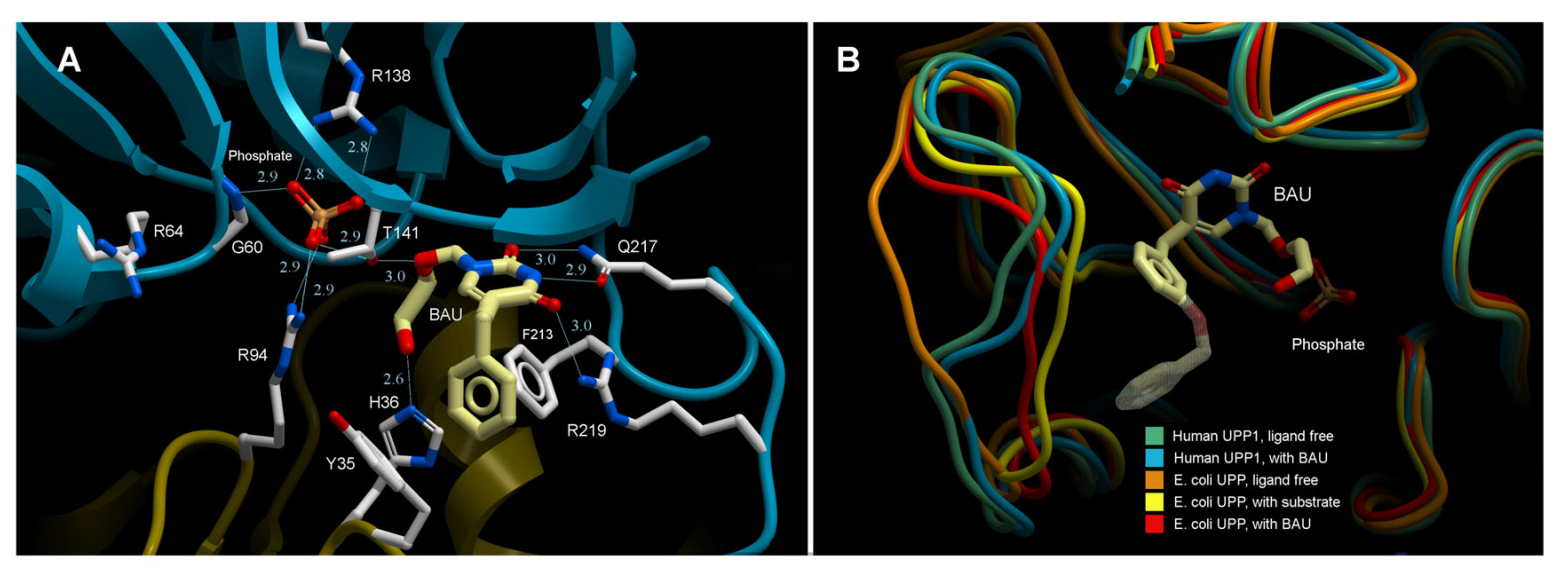
**BAU binding to hUPP1**. (A) BAU and phosphate coordination in hUPP1 is consistent with that previously observed in bacterial homologues with the notable exception that R64 (equivalent to R30 in *E. coli*) does not participate in phosphate binding, possessing instead a conformation that distances its guanidinium group from the negatively-charged oxygen atoms of the anion. (B) Shown is an alignment of three conformations of the *E. coli *enzyme with the two hUPP1 structures, focusing on the loop toward the back of the active site (human residues 278–284; boxed in Figure 2C). It has been hypothesized that in the bacterial enzyme, this loop undergoes an induced-fit conformational change upon ligand binding. In hUPP1, these residues are more ordered and conformationally restrained. They do not vary significantly in position between the BAU-bound and ligand-free structures, possibly due to the insertion of two additional residues within this region. These differences become increasingly important when considering the impacts of chemical modifications to the initial benzyl moiety of BAU, such as the characterized UPP inhibitor, 5-*m*-benzyloxybenzylacyclouridine (BBAU) shown (shaded).

Analysis of the catalytic active site reveals that BAU binds hUPP1 in a manner consistent with that previously characterized using EcUPP [7]. In actuality, all residues interacting with the natural uridine and phosphate ligands are strictly conserved between the two enzymes (Figure 2C). This fact was unclear prior to this study, as regions of these proteins between β2 and β3 lack sufficient sequence similarity for accurate alignment in the absence of additional structural data. Despite strict identity retention in ligand-binding residues, the coordination of the phosphate ion deviates from that observed in previous microbial structures (Figure 4A). Specifically, one of the three pocket arginine residues (Arg64 in hUPP1) is bent away from the anion, leaving only two coordinating guanidinium groups, one from each subunit of the dimer, to bind the substrate. The reason for the unexpected conformation of this arginine is not immediately apparent, as the backbone Cα of this residue is in an equivalent location with respect to the phosphate molecule as in other structurally analyzed UPPs. However, in the ligand-free structure of hUPP1 this residue is found to coordinate a sulfate ion from the crystallization liquor at a position 4.4 Å removed from the catalytic phosphate binding pocket. This finding may imply that in the human enzyme this residue has been adapted to ligand recruitment from the cytoplasm rather than ligand binding in the active site.

Additional differences between the active sites of human and bacterial enzymes become apparent when the analysis is extended to regions of these proteins contacting chemical modifications of acyclouridine analogues such as BAU. The benzyl ring of this molecule is π-stacked on one side by Tyr35, a residue that is conserved as phenylalanine in microbes. More significantly, the edge of this benzyl moiety interacts with a loop region that is structured very differently in hUPP1 than in EcUPP. In structures of the *E. coli *enzyme, the equivalent loop appears highly flexible, to the point of frequently being too disordered for modelling. These studies concluded that this loop undergoes an 'induced-fit' conformational change upon ligand or inhibitor binding (Figure 4B) [6]. In contrast, the same loop in hUPP1 is rigid with relatively lower thermal factors and remains structurally unaltered by BAU binding. This stability is likely the result of the insertion within this loop in hUPP1 of two additional residues as compared to bacterial variants (Figure 2C). These structural differences have major implications toward understanding how various other existing or theoretical acyclouridine derivatives will interact with the human enzyme.

### Ligand induced motion between hUPP1 domains

**Figure 5:**
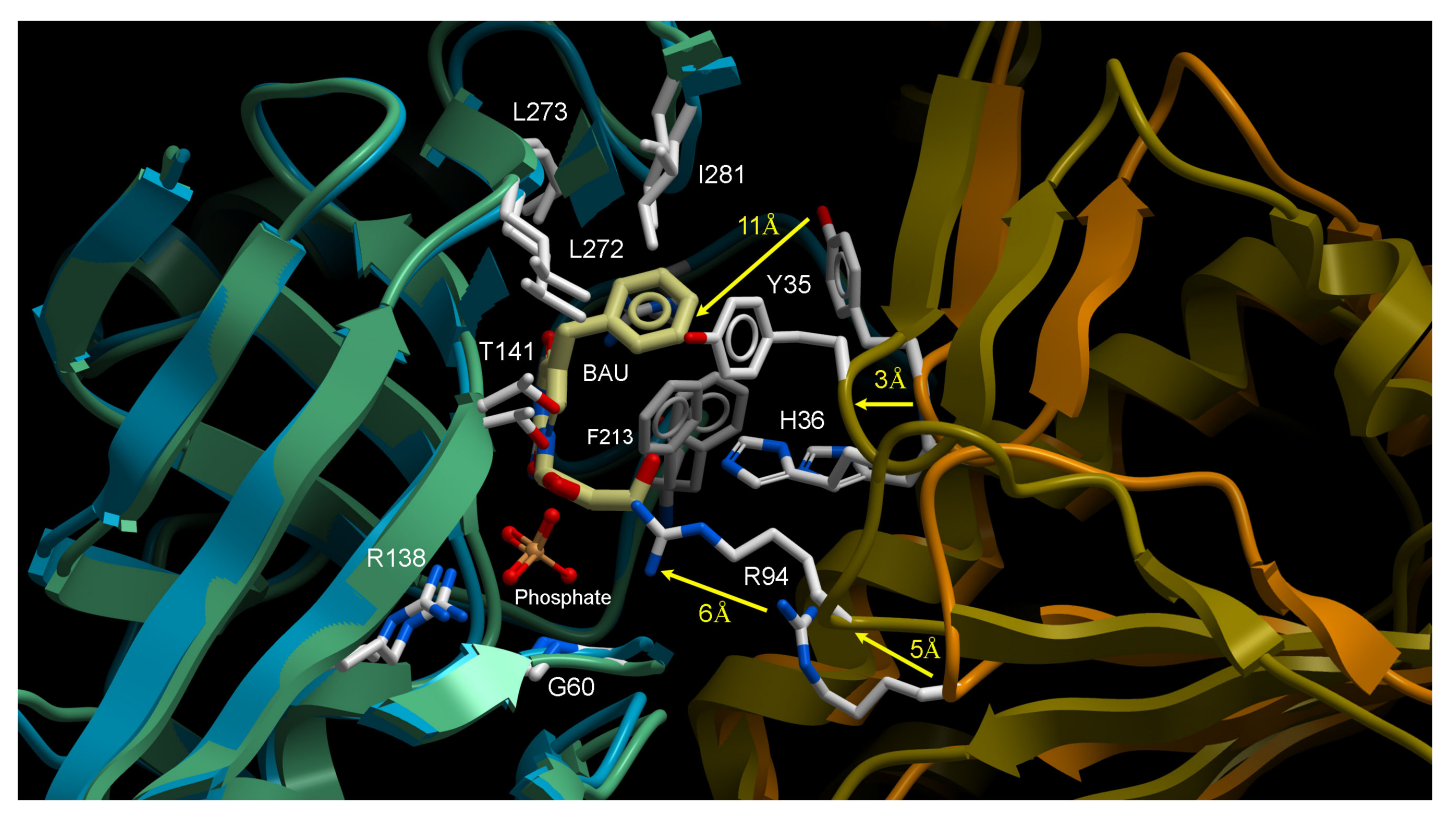
**Ligand mediated inter-domain motion of hUPP1**. While the structural conformations of monomers of hUPP1 vary only subtly between BAU-bound and ligand-free forms, there is substantial movement between the two domains upon inhibitor binding, as illustrated here. In the presence of BAU, phosphate coordination is also promoted within the substrate pocket, despite its absence in purification or crystallization solutions. The presence of both molecules within the ligand pocket causes the enzyme to dramatically close the active site, resulting in backbone carbon motion of 3–5 Å and individual residue repositioning of over twice that magnitude. The open state seen for the ligand-free structure of hUPP1 has not been previously observed in bacterial homologues, possibly because the hexameric assembly of these microbial enzymes restricts their range of inter-domain motion.

Determination of both ligand-free and BAU-bound forms of hUPP1 allows direct analysis of the conformational changes induced in the enzyme upon substrate binding. To this end, there are only subtle differences in the fold and residue orientation between overlaid monomers of hUPP1. However, there is a dramatic inter-domain motion between monomers leading to 3–5 Å changes in the relative positioning of the loops undergoing the greatest movement (Figure 5). This hinge-type conformational change results in a closure of the active site around its substrates and appears to be driven by the formation of interactions between Arg94 and phosphate, His36 and the ribose sugar group, and Tyr35 and the nucleobase. It is noteworthy that this domain motion has not been observed in any of the previously determined microbial structures despite multiple ligand-free structures, suggesting that the human enzyme is more mobile than its bacterial homologues.

## Discussion

Human uridine phosphorylase 1 has been the molecular target for the design of specific inhibitors intended to boost endogenous uridine levels for the purpose of rescuing normal tissues from the toxicity of fluoropyrimidine nucleoside chemotherapeutic agents. The structures reported here reveal significant differences between hUPP1 and previously characterized microbial UPPs that have meaningful implications toward the rational design of novel reagents with improved potency. In contrast to expectations, hUPP1 is dimeric, having lost the higher order assembly (trimer-of-dimers) that results in hexameric rings in more primitive UPPs. Possibly as a direct consequence, hUPP1 has two major architectural modifications, both of which serve to increase the contact surface area between domains. These alterations likely increase the stability of dimer association, potentially as compensation for stability lost on dissolution of the larger ring complex.

Another implication of the loss of hexameric structure appears to be that the human enzyme possesses greater inter-domain flexibility than its microbial counterparts. While the ability of enzymes to 'breathe' to facilitate substrate and product exchange is a common phenomenon, it has not been observed in previous studies of UPPs. Whether this inter-subunit motion has a meaningful affect on the kinetics and catalytic turnover rates of hUPP1, in comparison to EcUPP, remains to be investigated. However, the 'open' conformation of hUPP1 provides the opportunity to potentially develop a novel class of allosteric inhibitors of this enzyme that lock the protein in a functionally disabled form, with its catalytic residues too separated to lyse substrates.

## Conclusion

In this report, we present the first structures of human uridine phosphorylase 1. The specific biochemical features of the human version of this ubiquitous enzyme revealed by these studies offer improved understanding of how clinically-evaluated specific inhibitors, such as BAU, bind to and inactivate this protein. The molecular details regarding the residues of hUPP1 lining the binding pocket for such acyclouridine derivatives offer clear approaches to improving the specificity of these compounds to the human enzyme. Previous studies have successfully increased the affinity of acyclouridine analogues to hUPP1 through the creation of more hydrophobic variations of BAU such as 5-*m*-benzyloxybenzylacyclouridine [16]. Given the recent revelation that BAU may have cross-reactivity to other human enzymes such as aldehyde oxidase [18], designing rational alterations in the moieties extending beyond the first benzyl ring may result in substantial improvements in both compound activity and selectivity. Extremely high affinity inhibitors of human PNP (7 p*M*) have been obtained by using transition state mimetics derived from immucillins [19]. With the conservation of active site structure and catalytic mechanism among all NP-I family members, similarly potent antagonists of hUPP1 should be creatable. When combined with strategically selected structure-based modifications to optimize specificity for this enzyme, there is a strong potential to develop improved pharmaceuticals for incorporation into novel chemotherapeutic regimens with increased efficacy and reduced toxicity.

## Methods

### Protein production and purification

Production and isolation of hUPP1 was conducted as previously reported [20] and followed standard laboratory protocols for recombinant bacterial protein expression and purification. In brief, pQE plasmid containing an N-terminally six histidine-tagged construct of the enzyme was transformed into BL21(DE3) *E. coli*. Freshly transformed colonies were cultured in Terrific Broth and induced with 0.1 mM isopropyl-β-D-thiogalactopyranoside (IPTG) at an O.D. of 1.0. Growth was continued overnight at 18°C. Cells were harvested and resuspended in 50 mM Tris buffer pH 8.0, 300 mM KCl, 10% glycerol with 20 mM imidazole. The bacteria were then disrupted by sonication on ice and membranes with other insoluble material were pelleted by high speed centrifugation (100,000 × g). Recombinant hUPP1 was subsequently purified from the resulting supernatant using Ni-NTA affinity chromatography and batch eluted with 500 mM imidazole added to the sonication buffer above. Further purification was conducted using gel filtration chromatography over Superdex 200 resin equilibrated in 300 mM KCl, 50 mM Tris buffer pH 8.0 with 1 mM Tris (2-carboxy-ethyl) phosphine (TCEP). The final sample was verified to be homogenous by SDS-PAGE experiments and used directly for crystallization or biochemical analysis, as it was discovered that reduction of the salt concentration of the buffer below 250 mM at any point during the protein preparation process led to protein aggregation and precipitation.

### Crystallization

Large scale preparations of hUPP1 recombinant protein provided the starting material for initial crystallization trials. Purified hUPP1 at 4 mgs/mL was subject to crystal screening utilizing the JCSG+ crystallization screen (Qiagen) with supplementation of potential ligands BAU, uridine and phosphate. Initial promising leads with BAU containing 25% PEG 3350 and Bis-Tris buffer pH 5.5 were optimized to produce large (> 100 microns/dimension) crystals. Crystals would grow in the same condition in two morphologies: rods and pyramids; however, the triganol crystals invariably possessed high mosaicity and low resolution diffraction characteristics. The largest and best diffracting rod crystals were grown in 17% PEG 3350, 100 mM Bis-Tris buffer pH 5.5, 300 mM KCl, 30 mM MgCl_2 _with 1 mM BAU added to the protein (3 mgs/mL). Crystals were frozen by submersion in liquid nitrogen after a few seconds incubation in cryoprotectant containing the above constituents supplemented with 23% ethylene glycol. The ligand-free crystal form of hUPP1 was found in conditions optimized to 1.2 M (NH_4_)_2_SO_4_, 100 mM Bis-Tris buffer pH 5.5, with 1–2% MPD and protein concentration at 2–3 mgs/mL. Crystals could be grown to very large diamond-shaped proportions, exceeding 300 microns on each edge. These crystals were frozen by submersion in liquid nitrogen after a few seconds incubation in cryoprotectant containing the above constituents supplemented with 25% glycerol.

### Data collection/processing and structure determination

Data was collected at SSRL beamlines 7-1 and 9-1 as summarized in Table 1. Complete, high quality datasets to 1.9 Å and 2.3 Å resolution were obtained for the BAU-bound and ligand-free crystal forms, respectively. Collected data was processed and reduced by the HKL2000 package with Denzo and Scalepack [21]. The higher resolution crystal is of the orthogonal space group P2_1_2_1_2_1 _with low mosaicity. The other crystal form belongs to the face-centered cubic space group F4_1_32. Molecular replacement phasing of the data obtained on hUPP1 with BAU was successful using Molrep [22] with dimeric homology models of hUPP1, based on the BAU-bound *E. coli *UPP structure (PDB ID: 1U1C) [7], modified by Swiss-Model [23]. Solution phases were sufficient to resolve density for the unmodelled BAU ligand and other unmodelled residues. The initial model was rebuilt after phases were obtained using ARP/wARP [24]. Rounds of model building and refinement were performed using Coot [25] and Refmac [22]. Due to a lack of electron-density, the first 15 residues of hUPP1 and the N-terminal cloning artifact residues 'MRGSHHHHHHGSPGLQEF' were not built. Additionally, for the same reason the final two C-terminal residues could not be modelled in the BAU-bound structure. Medium non-crystallographic symmetry restraints (between 4 chains) were retained for the loop residues 79–84 in the BAU-bound structure due to the low quality of the electron-density map in this region of the protein. The BAU-bound model includes both a BAU molecule and a phosphate ion per protein chain as ligands that could be clearly built into the electron density. The native hUPP1 structure was completed with a sulfate ion (coordinated by Arg64) and a cation (modelled as magnesium based on interatomic distances) positioned at a crystal contact point between the side chain oxygen atoms of Asp212 and Ser116 of a symmetry-related chain. The final structures were refined with Refmac to an R_factor_/R_free _of 20.5%/25.1% respectively for the BAU-bound structure, and 20.4%/22.1% for the ligand-free structure of the enzyme, with approximately 92% of residues in most favorable regions of the Ramachandran plot as analyzed by Procheck [26]. The models were further validated using Molprobity [27], scoring in the 91^st ^and 97^th ^percentile, respectively. Figures were rendered using ICM Browser-Pro (Molsoft). The atomic coordinates and structure factors have been deposited in the Protein Data Bank (3EUF and 3EUE).

### Multi-angle light scattering analysis

Recombinant hUPP1 was analyzed using size exclusion chromatography over a calibrated Superdex 200 column on an Äcta Basic FPLC (GE) with an in-line MiniDAWN TREOS light scattering detector (Wyatt Technology) for in solution characterization of absolute molecular weight, size, and quaternary assembly, in combination with an Optilab rEX refractive index detector, in accordance with the Wyatt manual. The running buffer was comprised of 300 mM KCl, 50 mM Tris buffer pH 8.0, and 1 mM TCEP.

## Authors' contributions

TPR designed the study; supervised protein production, purification and crystallization; conducted X-ray data collection and processing; built and refined the structural models; drafted the manuscript; and prepared the figures. SC assisted in protein preparation and purification and conducted crystal screening of BAU-bound hUPP1. MF conducted crystal screening and optimization for ligand-free hUPP1. GP participated in the coordination of the study and helped edit the manuscript. All authors read and approved the final manuscript.
